# Effects of *in vitro* digestion on protein degradation, phenolic compound release, and bioactivity of black bean tempeh

**DOI:** 10.3389/fnut.2022.1017765

**Published:** 2022-10-12

**Authors:** Kun Wang, Yongjiao Gao, Jing Zhao, Yue Wu, Jingchen Sun, Guangcai Niu, Feng Zuo, Xiqun Zheng

**Affiliations:** ^1^College of Food Science, Heilongjiang Bayi Agricultural University, Daqing, China; ^2^National Coarse Cereals Engineering Research Center, Daqing, China; ^3^Engineering Research Center of Processing and Utilization of Grain By-products, Ministry of Education, Daqing, China

**Keywords:** black bean, tempeh, antioxidant, ACE-inhibitory activity, bioaccessibility

## Abstract

The nutritional value and bioactivity of black beans are enhanced when fermented as tempeh, but their bioaccessibility and bioactivity after ingestion remain unclear. In this study, black bean tempeh and unfermented black beans were digested *in vitro* and changes in protein degradation, phenolic compound release, angiotensin I-converting enzyme (ACE)-inhibitory activity, and antioxidant activity between the two groups were compared. We observed that the soluble protein content of digested black bean tempeh was generally significantly higher than that of digested unfermented black beans at the same digestion stage (*P <* 0.05). The degree of protein hydrolysis and the content of <10 kDa peptides were also significantly higher in the digested black bean tempeh than in digested unfermented black beans (*P* < 0.05). SDS-polyacrylamide gel electrophoresis (SDS-PAGE) and reversed-phase high-performance liquid chromatography (RP-HPLC) analysis showed that most macromolecular proteins in tempeh had been degraded during fermentation and more of the small peptides were released from black bean tempeh during digestion, respectively. Compared to that of the unfermented black beans, the level of ACE inhibition of black bean tempeh was lower, but this significantly increased to 82.51% following digestion, closing the gap with unfermented black beans. In addition, the total respective levels of phenolics, flavonoids, and proanthocyanidins released from black bean tempeh were 1.21, 1.40, and 1.55 times those of unfermented black beans following *in vitro* digestion, respectively. Antioxidant activity was also significantly higher in digested black bean tempeh than in digested unfermented black beans and showed a positive correlation with phenolic compound contents (*P* < 0.05). The results of this study proved that, compared to unfermented black beans, black bean tempeh retained protein and phenolic compound bioaccessibility and antioxidant activity and showed an improved ACE-inhibitory activity even after consumption.

## Introduction

Tempeh is a fermented bean-based product original from Indonesia that can be eaten as a staple food. Traditionally, tempeh is pie-shaped with white mycelia on the surface and it is obtained by soaking, peeling, cooking, and acid-adjusting ordinary soybeans, and finally fermenting them using *Rhizopus oligosporus* (1, 2). In recent years, various types of grains and legumes such as oats, barley, wheat, mung bean, broad bean, chickpea, and corn/soybean have also been used in the production of tempeh, further enriching its variety (3). Tempeh is a nutritious food, containing high amounts of dietary fiber, vitamin B12, folic acid, unsaturated fatty acids, isoflavones, and minerals, as well as a high protein content, making it an ideal meat substitute for vegetarians (2, 4, 5). In addition to its high nutritional value, tempeh also has a variety of physiological benefits such as antioxidation, lowering of blood lipid levels and blood pressure, tumor inhibition, prevention of atherosclerosis, and improvement of iron deficiency in anemia (3, 5, 6). Therefore, tempeh has cemented its position as a “superfood” in the modern society, in which the pursuit of high-quality nutrition has become increasingly important.

Besides being rich in high-quality protein, black beans [*Glycine max* (L.) merr.], otherwise known as black soybeans, also contain isoflavones, vitamin E, saponins, carotenoids, anthocyanins, and other active ingredients that grant them anti-inflammatory, antioxidant, anti-tumor, cholesterol-lowering, and analgesic benefits, and making them one of the black-colored foods with the highest nutritional and health care value (7–10). However, currently, the evaluation of the nutrition and health benefits of food is mainly performed through direct chemical analysis without considering the influence of bodily digestion upon human consumption. The conditions of human gastrointestinal digestion and organic extraction can differ greatly, and certain biologically active ingredients can undergo further degradation and metabolism under the action of digestive enzymes during the process of digestion, potentially affecting their biological activity following digestion. Giusti et al. (11) reported that only a fraction of the phenolic compounds from the coat and cotyledons of black beans was bioaccessible after simulated gastrointestinal digestion, and some phenolics were absent after small intestinal digestion due to their pH instability. Sancho et al. (12) investigated the effects of *in vitro* digestion on polyphenols and the antioxidant activity of black bean seed coats and found that the digestion reduced the levels of total phenols and anthocyanins but not the antioxidant activity. Meanwhile, López-Barrios et al. (13) observed that the anti-inflammatory activity of black bean protein isolates was reduced after simulated gastrointestinal digestion. Though the nutritional value and bioactivity of black beans are enhanced when fermented as tempeh (1, 14), it remains unclear whether the active ingredients of black bean tempeh can still retain their biological activity after gastrointestinal digestion *in vitro*.

Based on the previous research, this study simulated the buccal, gastric, and small intestinal digestion of black bean tempeh *in vitro* and investigated the protein degradation, phenolic release, angiotensin I-converting enzyme (ACE)-inhibitory activity, and antioxidant activity of black bean tempeh to assess the effects of digestion on its nutrient release and bioactivity. This study can serve as a theoretical reference for the development of black bean products with high nutritional value and bioactivity.

## Materials and methods

### Chemicals and materials

Black beans were purchased from Jiansanjiang Farm, Heilongjiang Province, China. Food-grade lactic acid was purchased from Zhengzhou Gaoyan Biotechnology Co., Ltd. (Zhengzhou, China). *R. oligosporus* starter powder was purchased from Nanjing Tianbeiren Fermentation Technology Co., Ltd. (Nanjing, China). Chromatography-grade methanol, trifluoroacetic acid (TFA), and acetonitrile were purchased from Merck (Darmstadt, Germany). Water-soluble vitamin E (Trolox), 1,1-diphenyl-2-picrylhydrazyl (DPPH), pepsin, trypsin, salivary amylase, angiotensin I-converting enzyme (ACE), and 2,2’-azino-bis(3-ethylbenzothiazoline-6-sulfonic acid) (ABTS) were purchased from Sigma-Aldrich Chemical Co., Ltd. (St. Louis, MO, USA). All other chemicals and reagents were of analytical grade and purchased from Sinopharm Chemical Reagents Co., Ltd. (Shanghai, China).

### Black bean tempeh production

Black beans were washed with clean water, soaked in a ratio of 1:3 beans to water (m/v) for 24 h at room temperature, peeled, and steamed until soft and without hard cores. After cooling to room temperature, 1.5% (w/w) lactic acid and 0.26% (w/w) *R. oligosporus* starter powder were added and stirred evenly, then placed in an 8 cm × 12 cm fermenting bag with holes (1.25 holes per square centimeter) and incubated at 35 °C for 39 h to obtain the finished product. Unfermented cooked beans served as the experimental control.

### *In vitro* digestion

The preparation of artificial saliva, gastric juice, and small intestinal juice, as well as the procedures for *in vitro* digestion, were performed according to the methods reported by Rui et al. (15) and Xing et al. (16) with a slight modification. Briefly, 10 g of fresh samples were weighed, and 40 ml of distilled water was added to homogenize the initial samples with a miniature beater. To simulate buccal digestion, 20 ml of 0.2 mg/ml salivary amylase (in 20 mM phosphate buffer at pH 7.0) was added to the initial sample, and the samples were put into a shaking water bath (SWB series; Biobase, Shandong, China) at 55 rpm and 37°C for 3 min. Then, 6 M HCl was used to adjust the pH to 2.0, and 30 ml of 3.2 mg/ml pepsin (in 0.1 M HCl) was added. The simulation of gastric digestion was performed at 55 rpm, 37°C for 1 h. Subsequently, the pH of the reaction was adjusted to 7.0 with 6 M NaOH, and then 20 ml of artificial bile acids (0.4 mg/ml sodium cholate in 10 mM phosphate buffer at pH 7.0) and pancreatic juice (0.4 mg/ml trypsin in 10 mM phosphate buffer at pH 7.0) were added, and the slurry was shaken at 37°C and 150 rpm for 120 min to simulate small intestinal digestion. At the end of each stage, the digestion solution was boiled for 5 min to terminate the enzymatic hydrolysis reaction, then distilled water was added for a constant volume of 130 ml. Next, the solution was centrifuged at 4°C and 12,000 r/min for 15 min, then the supernatant was collected and frozen at −20°C for later use. The samples were labeled according to the digestion stage and substrate; namely, P1 was the initial sample of unfermented black bean control, and P2–P4 were the control samples following the simulated buccal, gastric, and small intestinal digestion. P5 was the initial sample of black bean tempeh and P6–P8 were the tempeh samples following the simulated buccal, gastric, and small intestinal digestion.

### Soluble protein analysis

The soluble protein content was determined by the Bradford protein quantification assay using bovine serum albumin as the protein standard (17).

### Electrophoresis

SDS-polyacrylamide gel electrophoresis analysis was performed using a DYCZ-24D vertical electrophoresis unit (Baygene Biotech Co., Ltd., Beijing, China) coupled with a DYY-Ш-5 electric source (Beijing Liuyi Instrument Factory, Beijing, China). The loading volume was 25 μl. Separation gels consisted of a 5% polyacrylamide stacking gel and a 12% polyacrylamide resolving gel, and the voltages used for the gels were 60 and 120 V, respectively. A protein standard (14.4–116.0 kDa) was used as the molecular mass ladder.

### Determination of degree of hydrolysis

The *o*-phthaldialdehyde (OPA) spectrophotometric assay was used to determine the number of free amino groups and quantitatively analyze the degree of proteolysis (18). Three milliliters of OPA reagent was added to 400 μl of the solution under test. After mixing, the reaction was accurate for 2 min at room temperature, and the absorbance was measured at 340 nm wavelength using a SPECORD 210 Plus UV/VIS Spectrometer (Analytik Jena AG, Germany). Peptide bonds were quantified using 0.1 mg/ml serine standard solution. The degree of hydrolysis (DH)was calculated according to the formula (1):


(1) $$D\,H\%=\frac{\left(W_{s\,e\,r\,i\,n\,e-N\,H\,2}-\beta \right)}{\alpha \times H_{t\,o\,t}}\times 100$$

where


(2) $$W_{s\,e\,r\,i\,n\,e-N\,H\,2}=\frac{A_{s\,a\,m\,p\,l\,e}-A_{b\,l\,a\,n\,k}}{A_{s\,t\,d}-A_{b\,l\,a\,n\,k}}\times \frac{C_{s\,e\,r\,i\,n\,e-N\,H\,2}}{C_{p\,r\,o}}$$

and *W*_*serine–NH2*_ is the millimole number of peptide bonds of the sample protein (mmol/g), *A*_*sample*_ is the absorbance of the sample tube, *A*_*blank*_ is the absorbance of the blank tube, *A*_*std*_ is the absorbance of the serine standard tube, *C*_*pro*_ is the concentration of the sample protein (g/L), *C*_*serine–NH2*_ is the concentration of the serine standard solution (0.9516 mM), α and β are constants of 0.4 and 1, respectively, and the total peptide bond number (*H*_*tot*_ value) of black bean protein was 10.32 mmol/g.

### Measurement of peptide contents

Peptide contents were measured as previously reported (19). The solution under test was filtered using a filter membrane with an interception molecular weight of 10,000 Da (Millipore, USA), and then 50 μL of the filtrate was absorbed and reacted with 2 ml of OPA reagent for 2 min at room temperature. The absorbance was measured at a wavelength of 340 nm. A series of casein tryptone solutions at different concentrations were used as the standard for quantification. The results were expressed as casein tryptone equivalents (mg CTE per g fresh sample).

### Reversed-phase high-performance liquid chromatography

Reversed-phase high-performance liquid chromatography (RP-HPLC) analysis was performed using a 1260 Infinity II HPLC (Agilent, USA) equipped with a Supersil AQ-C18 reverse-phase column (250 mm × 4.6 mm, 5 μm, Dalian Elite Analytical Instruments, Dalian, China). After filtration using a 0.45-μm filter, 20 μl of sample was applied to the column. The column temperature was 25°C. The mobile phases were composed of solvent A (0.10% TFA in water, v/v) and B (0.10% TFA in acetonitrile, v/v). The elution program started at 5% B, was increased to 17% within 16 min and further to 95% within 2 min, then maintained at 95% for 2 min before being increased to 100% within 1 min and then maintained at 100% for 7 min. The flow rate was 0.80 ml/min and the elution was monitored at 282 nm using a diode-array detector.

### Analysis of angiotensin I-converting enzyme-inhibitory activity

An appropriate volume of the solution under test was mixed with 0.1 M boric acid buffer (pH 8.3) in equal volume, and the supernatant was collected by centrifugation at 12,000 rpm, 4°C for 15 min after standing for 1 h. According to a previous method reported by Zhu et al. (20), the determination of the ACE-inhibitory activity of samples was done through monitoring the formation of hippuric acid (HA) from Hippuryl-His-Leu (HHL) as the reaction substrate. Briefly, 10 μl of supernatant was mixed with 50 μl of 2.17 mM HHL and 10 μl of ACE (100 mU/ml). After 30 min of shock reaction at 37°C, the reaction was terminated by adding 85 μl of 1 M HCl. The reaction solution was filtered using a 0.45-μm filter membrane and analyzed using Agilent 1260 Infinity II HPLC. The sample (20 μl) was injected into a Supersil AQ-C18 column (250 mm × 4.60 mm, 5 μm, 25°C, Dalian Elite Analytical Instruments, Dalian, China) and eluted with a 50% methanol solution (v/v) containing 0.1% TFA at a flow rate of 0.50 ml/min. The detection wavelength was 228 nm.

The ACE inhibition rate (IR) of the sample was calculated according to formula (3):


(3) $$IR\left(\%\right)=\frac{A_{1}-A_{2}}{A_{1}-A_{3}}\times 100$$

where *A*_1_ is the peak area of the reaction system when boric acid buffer is used instead of sample, *A*_2_ is the peak area of the sample reaction system, and *A*_3_ is the peak area of the reaction system without ACE.

### Determination of total phenolic content

The total phenolic content (TPC) was measured using the Folin-Ciocalteu method as described by Tao et al. (21). Folin-Ciocalteu’s reagents (0.5 ml) and 2.3 ml of deionized water were added to 200 μl of the solution under test and the mixture was incubated for 1 min. Then, 2 ml of 7.5% (w/v) Na_2_CO_3_ was added and the mixture was subjected to a dark reaction for 2 h at room temperature. After the reaction was complete, the absorbance at 760 nm was measured with deionized water as the blank control. TPC was expressed as gallic acid equivalents (mg GAE per g fresh sample).

### Determination of total flavone content

The total flavone content (TFC) was determined by referring to the method of Sandhu and Punia with a slight modification (22). A 60% ethanol solution (4.4 ml) and a 5% (g/ml) NaNO_2_ solution (0.3 ml) were added to 0.6 ml of the solution under test, then mixed and left to rest for 6 min. Next, 0.3 ml of 10% Al(NO_3_)_3_ solution was added and the mixture was left to rest for another 6 min. Finally, 4.0 ml of 1 M NaOH solution was added, and the volume was fixed to 10 ml with 60% ethanol solution. The absorbance at 510 nm was measured with deionized water used as the blank control. The TFC was expressed as rutin equivalents (mg RE per g fresh sample).

### Determination of proanthocyanidin content (PC)

The proanthocyanidin content (PC) was determined according to the method reported by Saifullah et al. (23). The mixture of 1 ml of the solution under test, 3 ml of 3% (g/ml) vanillin-methanol solution, and 1.5 ml of concentrated hydrochloric acid was placed in a water bath at 30 °C in the dark for 15 min and then the absorbance at 500 nm was measured with methanol as blank control. The PC was expressed as catechin equivalents (mg CE per g fresh sample).

### Antioxidant activity analysis

The DPPH radical scavenging activity was determined according to the method described by Tian et al. (24). The ABTS^+^ radical scavenging ability was determined according to the method of Dong et al. (25). The hydroxyl radical scavenging activity and ferric reducing antioxidant power (FRAP) were determined according to the method of Wang et al. (26). Trolox was used as the positive control and FRAP was expressed as FeSO_4_ equivalents (μmol FeSO_4_ equivalents per g fresh sample).

### Statistical analysis

All assays were performed in triplicate, and the results are presented as the mean ± standard deviation (SD). Statistical analysis was performed using the SPSS 17.0 software. One-way ANOVA and Duncan’s test were used to analyze significant differences in the means at the 0.05 level.

## Results and discussion

### Soluble protein contents

**FIGURE 1:**
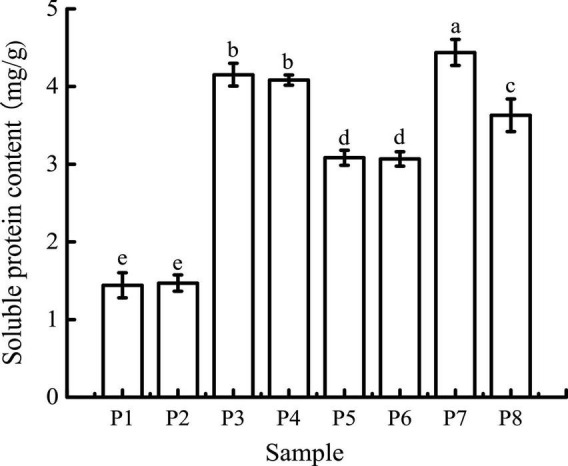
Soluble protein contents of samples in different simulated digestion stages. P1 was the initial sample of unfermented black bean control and P2–P4 were the control samples following the simulated buccal, gastric, and small intestinal digestion. P5 was the initial sample of black bean tempeh and P6–P8 were the tempeh samples following the simulated buccal, gastric, and small intestinal digestion, similarly hereinafter. Data with different lowercase letters indicate significant differences (*P* < 0.05).

As shown in Figure 1, the average soluble protein contents of black bean tempeh and unfermented black beans were 3.08 and 1.44 mg/g, respectively, indicating that *R. oligosporus* fermentation evidently induced the hydrolysis of black bean proteins. The soluble protein contents of both samples were largely unchanged after buccal digestion. This is because artificial saliva lacks a protein digestive enzyme system, and salivary amylase mainly acts on soluble starch such as amylose and glycogen; therefore, it has no obvious effect on protein structure. In the subsequent stage of gastric digestion, the presence of pepsin, a strong acid environment, and continuous mechanical shaking causes the disintegration of the protein network and promotes the release of soluble proteins (27). The average soluble protein contents of both black bean tempeh and unfermented black bean samples were significantly increased to 4.44 and 4.15 mg/g, respectively. The soluble protein content of black bean tempeh samples was still significantly higher than that of unfermented black bean samples (*P* < 0.05), which may be because a large degree of degradation of black bean tempeh proteins had occurred during the fermentation stage, causing the spatial structure of the proteins to be relatively relaxed, exposing a large number of digestive enzyme action sites, thus making them more conducive to pepsin digestion. Following small intestinal digestion, the soluble protein content of unfermented black beans was chiefly unchanged, but that of black bean tempeh was significantly lower than that of the tempeh sample in the previous stage of digestion (*P* < 0.05). This likely happened because partial soluble proteins released in the early stage of digestion were further degraded into peptides or free amino acids, which are not detected by Coomassie brilliant blue dye (17). The difference in the outcome of trypsin hydrolysis during small intestinal digestion also suggested that *R. oligosporus* fermentation had a considerable effect on the amino acid composition of black bean protein products after *in vitro* gastrointestinal digestion, imparting a positive effect on digestion.

### Electrophoresis

**FIGURE 2:**
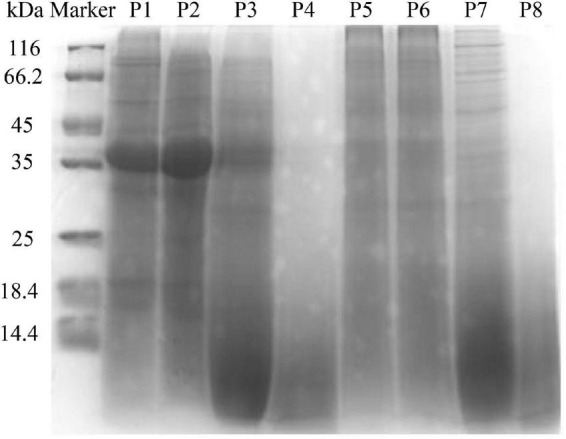
SDS-polyacrylamide gel electrophoresis (SDS-PAGE) patterns of samples in different simulated digestion stages.

The molecular weight distribution of unfermented black bean and black bean tempeh proteins was investigated by SDS-PAGE during *in vitro* digestion. In Figure 2 it can be seen that the protein bands of P1 were mainly concentrated between 35 and 45 kDa, and several clear bands also appeared in the >45-kDa region. However, the intensity of the P5 band was lighter and diffusely distributed within the whole lane. These results indicated that *R. oligosporus* fermentation mainly induced the hydrolysis of 35–45-kDa proteins in black beans. The electrophoretic bands of P2 and P6, which represented protein samples at the buccal digestion stage, remained unchanged compared with the samples at the previous stage, indicating that buccal digestion had no obvious effect on the proteins, consistent with the previous results of soluble protein content analysis. The hydrolysis of the sample proteins mainly occurred at the gastric digestion stage. After gastric digestion, the electrophoretic bands of both samples changed significantly. The intensity of the protein bands of P3 within the >35-kDa region, especially the bands between 35 and 45 kDa, became evidently lighter, while the intensity of the protein bands of P3 within the <14.4-kDa region was significantly higher. Meanwhile, the P7 bands migrated further into the <14.4-kDa region. In the final stage of small intestinal digestion, the P4 and P8 bands moved further down, became lighter in intensity, and began to disappear, indicating that the proteins had been further degraded into low molecular fragments that could not be detected by SDS-PAGE (16). Overall, the patterns of electrophoretic protein bands between unfermented black bean samples and black bean tempeh samples during *in vitro* digestion were essentially similar and consequently displayed similar effects of digestion. However, it is worth noting that unlike the macromolecular proteins in the unfermented black bean samples, most of those in black bean tempeh samples had been hydrolyzed to small molecules before digestion, thus reducing the workload of protein digestion.

### Degree of hydrolysis and peptide contents

**FIGURE 3:**
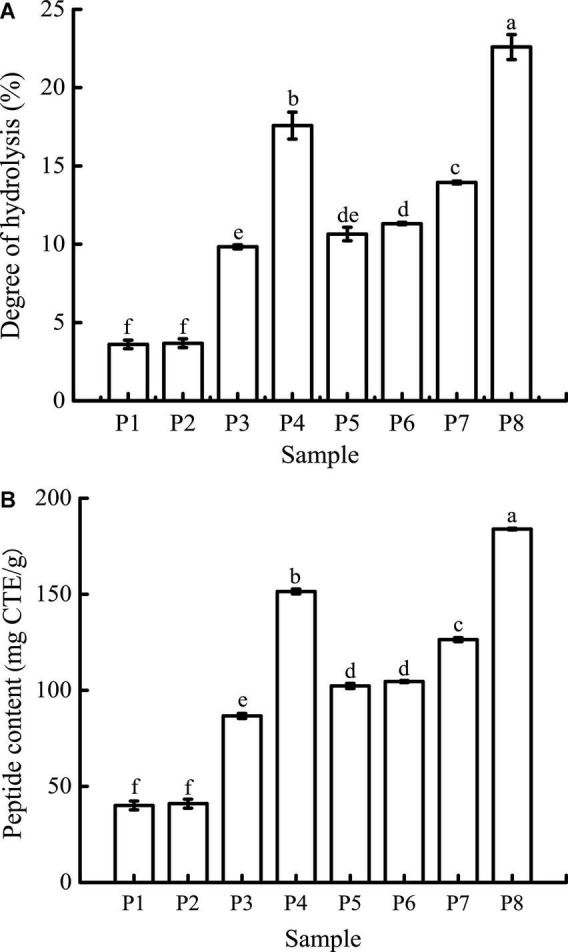
Degree of hydrolysis (DH) **(A)** and peptide contents **(B)** of samples in different simulated digestion stages. Data with different lowercase letters indicate significant differences (*P* < 0.05).

As can be seen in Figure 3A, the DH of unfermented black bean and black bean tempeh proteins were 3.60 ± 0.27% and 10.65 ± 0.43%, respectively. Buccal digestion did not affect the proteolysis degree of samples, but the DH of each sample showed a continuous and obvious upward trend in the gastrointestinal digestion stage. This was due to the proteins being successively hydrolyzed by pepsin and trypsin in the gastric and small intestinal fluids, leading to the release of a large number of peptides and free amino acids. After digestion, the DH of samples increased to 17.57 ± 0.86% and 22.60 ± 0.8%, respectively. The DH of tempeh proteins was 0.29 times higher than that of unfermented black bean proteins. This fully indicated that the tempeh proteins were hydrolyzed more thoroughly than the unfermented black bean proteins after digestion *in vitro*, making them more conducive to subsequent absorption and utilization. As can be seen in Figure 3B, the content of peptides below 10 kDa in the unfermented black bean samples and black bean tempeh samples did not change significantly after buccal digestion, but they progressively increased significantly (*P* < 0.05) after the gastric and small intestinal digestion stages, with the unfermented samples and tempeh samples increasing from 40.03 ± 2.30 mg CTE/g and 102.26 ± 1.29 mg CTE/g to 151.44 ± 1.25 mg CTE/g and 183.95 ± 0.43 mg CTE/g, respectively. At the same digestion stage, the peptide content of tempeh was generally significantly higher than that of unfermented black beans. The production of small peptides (below 10 kDa) may confer better bioactivities such as antioxidation and anti-tumor activities, as well as blood pressure-lowering and anti-inflammation properties to black bean tempeh (17).

### Reversed-phase high-performance liquid chromatography (RP-HPLC)

**FIGURE 4:**
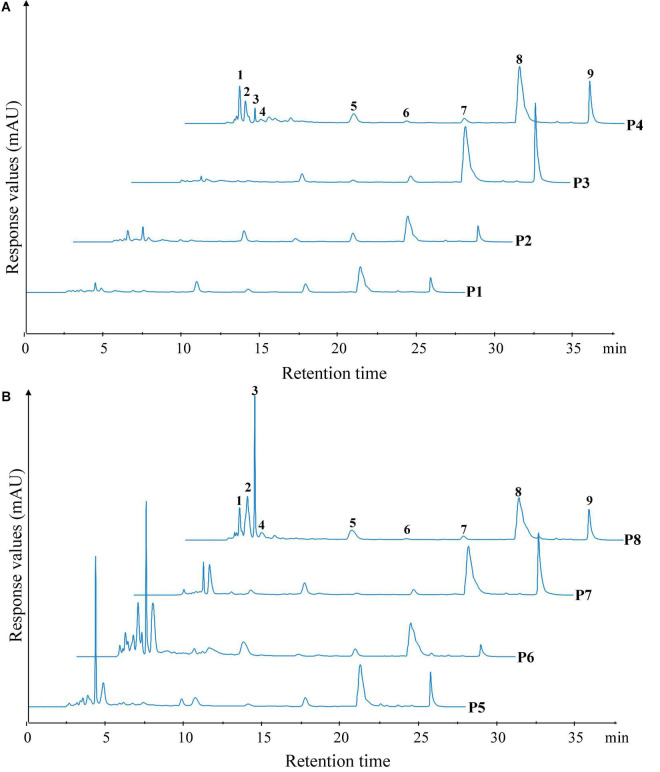
Reversed-phase high-performance liquid chromatography (RP-HPLC) patterns of unfermented black bean **(A)** and black bean tempeh **(B)** samples in different simulated digestion stages. Peaks with larger areas or significant changes were successively labeled 1–9 according to their order.

The peptide profiles of unfermented black beans and black bean tempeh were analyzed by RP-HPLC at different stages of digestion. Differences in hydrophobicity can lead to differences in the binding ability of peptides and proteins to the reversed-phase column material, and they can be eluted sequentially under suitable conditions (18). The results are shown in Figure 4. Within the retention time from 3.5 to 28 min, the RP-HPLC chromatograms of all the samples with different digestion treatments showed a total of nine chromatographic peaks with larger areas or significant changes, which were successively labeled 1–9 according to their order. There was no significant change in the species and area of main peaks in the buccal digested samples (P2/P6) of unfermented black beans and black bean tempeh compared with the same groups of samples prior to digestion (P1/P5). The stages with a greater influence on peak output were mainly gastric digestion and small intestinal digestion. The respective areas of peaks 8 and 9 of gastric digested samples from unfermented black beans (P3) were 2.34 and 5.20 times those of buccal digested samples from unfermented black beans (P2), while the respective areas of peaks 6, 8, and 9 of gastric digested samples from black bean tempeh (P7) were 1.11, 1.32, and 5.40 times those of buccal digested samples from black bean tempeh (P6). However, the peak areas numbered 1–4 in both samples decreased significantly after gastric digestion. The eluting components appearing in the early stage should be the peptide segments with very low molecular weight (≤22 kDa) and high hydrophilicity (28), which indicates that gastric digestion mainly promotes the hydrolysis of hydrophilic, small-molecule peptides and the release of more hydrophobic, large-molecule peptides. This is consistent with the result that gastric digestion caused a significant increase in the DH of samples. The areas of peaks 1–3 and 5 in the small intestinal digestion samples (P4/P8) were significantly increased compared with those of the samples in the previous stage. Most notably, the areas of peak 1 from the samples of unfermented black beans and black bean tempeh were 38.82 and 45.13 times those of the gastric digestion samples, respectively. However, the areas of peak 9 in the small intestinal digestion samples were significantly reduced, suggesting that these newly added small peptides may come from the degradation of elution components in the later stage. In addition, the total area of early elution peaks of black bean tempeh samples, both at the initial stage and at the end of digestion, was much higher than those of unfermented black bean samples, and the respective areas of peaks 2–5 of P8 were 1.92, 7.68, 1.03, and 0.30 times higher than those of the peaks of P4. However, the areas of peak 8 and peak 9 were lower than those of unfermented black bean samples. Combined with the previous soluble protein content and DH analysis results, we speculate that this was potentially because the digestion limit of black bean tempeh proteins under these conditions had already been reached, so the small intestinal digestion stage was unable to extract any more peptide segments. In summary, *in vitro* digestion caused the degradation of black bean proteins and the release of polypeptides, and *R. oligosporus* fermentation promoted the release of a higher number of small peptides during the process of digestion, which could improve the absorption and utilization of proteins in black bean tempeh by the human body.

### Angiotensin I-converting enzyme-inhibitory activity

**FIGURE 5:**
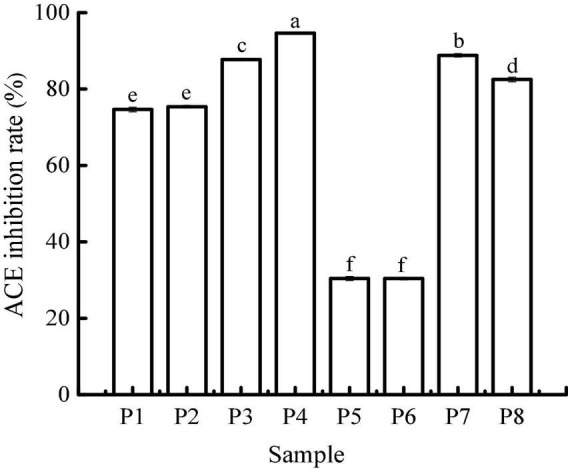
Angiotensin I-converting enzyme (ACE)-inhibitory activity of samples in different simulated digestion stages. Data with different lowercase letters indicate significant differences (*P* < 0.05).

Angiotensin I-converting enzyme regulates arterial blood pressure by converting inactive angiotensin I into angiotensin II, which has a strong vasoconstrictor effect and causes a rise in blood pressure (29). Therefore, the use of natural ACE inhibitors can effectively reduce blood pressure to a certain extent and avoid the potential risks caused by synthetic drugs. The ACE-inhibitory activity of unfermented black beans and black bean tempeh in different digestion stages is shown in Figure 5. The inhibition rate of ACE in unfermented black bean samples showed no change during buccal digestion but continued to increase after gastrointestinal digestion and ultimately reached 94.67 ± 0.07%. The change in the ACE inhibition rate in black bean tempeh samples was similar to the change observed in unfermented black bean samples during buccal and gastric digestion, but this trend decreased slightly after small intestinal digestion. Overall, the inhibition rate of black bean tempeh samples was also greatly improved, from the original 30.63 ± 0.67% up to 82.51 ± 0.63%. It is worth noting that the initial ACE-inhibitory activity of black bean tempeh was much lower than that of unfermented black beans. At first glance, this observation seemed to be inconsistent with the results above showing that fermentation leads to the increase of DH and peptide release of black bean proteins, especially small peptides, which are believed to have a higher ACE-inhibitory activity (30). However, there is no conclusive study on the influence of fermentation on the ACE-inhibitory activity of food. A study by Wu et al. (18) showed that the ACE-inhibitory activity of oats fermented by *Rhizopus oryzae*, either alone or in conjunction with *Lactobacillus plantarum* B1–6, was higher than that of unfermented oats. Rui et al. (29) also found that lactic acid bacteria fermentation could improve the release of ACE-inhibitory peptides from navy bean milk. However, a previous study by Nielsen et al. (31) showed that 13 lactobacillus strains had different effects on the ACE-inhibitory activity of yogurt. Although most strains contributed to the increase of the inhibitory activity, *Lactobacillus helveticus* fermentation could lead to a decrease in the ACE-inhibitory activity of yogurt (pH 3.5). The ACE-inhibitory activity of peptides is influenced by multiple factors, including molecular weight distribution and structural features such as terminal amino acid residues (30, 32). The differences in microbial restriction sites and the structure of raw proteins may influence the ACE-inhibitory activity of fermented products, which may be the reason for the lower initial ACE-inhibitory activity of black bean tempeh in this study. However, interestingly, *in vitro* digestion significantly increased the ACE-inhibitory activity of black bean tempeh by 1.69 times, greatly closing the gap with unfermented black beans. Jakubczyk et al. (33) fermented pea seeds with *L. plantarum* 299V for different times and at different temperatures and found that none of the fermented samples possessed ACE-inhibitory activity, though *in vitro* digestion subsequently induced the release of ACE-inhibitory peptides. Studies by Vermeirssen et al. (34) showed that *Saccharomyces cerevisiae* fermentation did not influence the ACE-inhibitory activity of whey protein under 28°C. The ACE-inhibitory activity of unfermented whey protein and pea protein *in vitro* digestion solution was even higher than that of the fermented sample. Therefore, they hypothesized that *in vitro* digestion was the predominant factor influencing the ACE-inhibitory activity.

### Phenolic compound contents

Bioaccessibility, defined as the potential for compounds to be released from the food matrix and be dissolved in the gastrointestinal tract during digestion, is one of the most important factors that determines the bioavailability of phenolic compounds. *In vitro* digestion models are often used to study the effects of digestion on these compounds to predict their release from the food matrix and assess their bioaccessibility (35).

**FIGURE 6:**
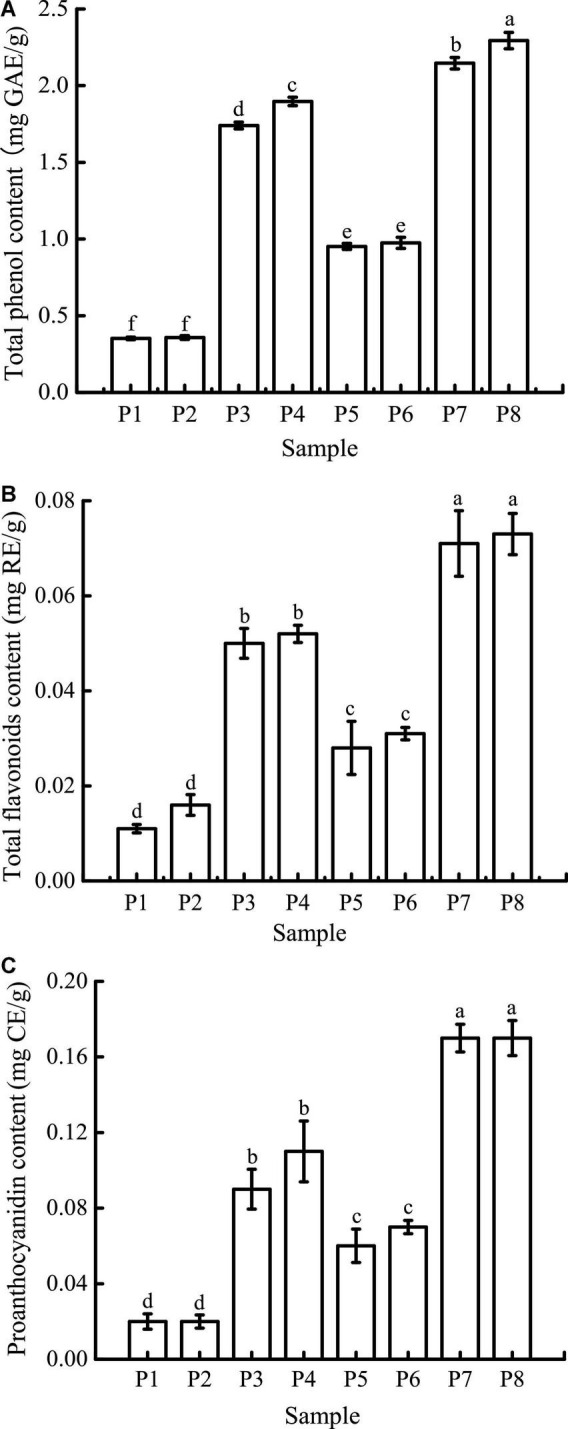
Total phenolic content (TPC) **(A)**, total flavone content (TFC) **(B)**, and proanthocyanidin content (PC) **(C)** of samples in different simulated digestion stages. Data with different lowercase letters indicate significant differences (*P* < 0.05).

The TPC at different digestion stages is shown in Figure 6A. The initial TPC of unfermented black bean samples and black bean tempeh samples were 0.35 ± 0.01 mg GAE/g and 0.95 ± 0.02 mg GAE/g, respectively. There were no significant changes in the TPC in both types of samples after buccal digestion, which might be due to the short interaction time with the buccal enzyme system. This implies that buccal digestion had little effect on polyphenol availability and was limited to high-carbohydrate foods, which explains why many studies did not carry out this step on phenolic compounds (36, 37). The TPC increased significantly after gastric digestion, most likely because plant polyphenols mainly exist in covalently bound states and thus are not easily released by conventional treatment. Extreme pH and the presence of digestive enzymes in the stomach enable them to be released from proteins (38, 39). After intestinal digestion, TPC was further increased and ultimately reached 1.90 ± 0.03 mg GAE/g and 2.29 ± 0.05 mg GAE/g in the unfermented black bean samples and tempeh samples, respectively. Similar results were reported by Scrob et al. (40) in their study, in which the TPC of dried fruits after gastrointestinal digestion was increased compared to the TPC of dried fruits after gastric digestion; according to the researchers, this phenomenon could be explained by the extra extraction time and the influence of intestinal digestive enzymes on the food matrix. However, studies with opposing results also exist in the literature. For example, *in vitro* gastrointestinal digestion was shown to greatly reduce the TPC of brown algae extract (41), and simulated gastric digestion led to a substantial reduction in the phenolic compound content of *Arbutus unedo* (42). The bioaccessibility of polyphenols is influenced by multiple factors, such as their own physicochemical properties, food matrix, interaction with other components, and the presence of cofactors or inhibitors (35). Therefore, we speculate that the composition of phenolic compounds in the food matrix might be the cause of these differences in the results.

As shown in Figure 6B, the initial TFC of unfermented black bean samples was 0.011 ± 0.001 mg RE/g, which was not significantly affected by buccal digestion. After gastric digestion, the TFC increased sharply and then remained basically unchanged after small intestinal digestion before finally reaching 0.052 ± 0.002 mg RE/g. The TFC change in the whole digestive process of black bean tempeh was the same as that of unfermented black beans, which increased from 0.028 ± 0.006 mg RE/g to 0.073 ± 0.004 mg RE/g, indicating that *in vitro* digestion promoted the release of flavonoids from black beans. Unlike TPC, small intestinal digestion failed to further improve the TFC of the samples. A similar trend was observed in the *in vitro* digestion of baobab fruit shell, where there was no significant difference in the TFC between samples undergoing gastric and small intestinal digestion (37). Meanwhile, Gawlik-Dziki et al. (43) found that the TFC of bread without or with the addition of 2.5% tartary buckwheat flavones was increased during *in vitro* digestion, but small intestinal digestion led to a significant decrease in the TFC of bread with the addition of 5% tartary buckwheat flavones. This implies that the food matrix as well as the flavonoid content may have an important effect on the release of flavonoids during *in vitro* digestion.

The trend in the change in PC was similar to that in TFC (Figure 6C). Gastric digestion is an important stage for PC increase, which is potentially due to the strong acid environment at this stage, thus promoting the hydrolysis of proanthocyanidins to isoforms such as proanthocyanidin trimers, tetramers, and pentamers (37). The final PC of unfermented black bean samples and black bean tempeh samples reached 0.11 ± 0.02 mg CE/g and 0.17 ± 0.02 mg CE/g, respectively. Ismail et al. (37) also found that the baobab fruit shell PC increased after gastric digestion, and what differed from our findings was that the PC continued to increase significantly after small intestinal digestion. They believed that the release of food matrix-binding proanthocyanidins catalyzed by trypsin or the depolymerization and release of insoluble proanthocyanidins caused by the rise of pH were the possible reasons for this result.

In conclusion, although the content of phenolic compounds in the digestion solution of unfermented black bean samples was also significantly increased during *in vitro* digestion, the release of phenolic compounds from black bean tempeh samples was generally significantly higher than that of unfermented black bean samples at the same digestion stage (*P* < 0.05). These results indicated that fermentation could improve the bioaccessibility of phenolic compounds in black beans. This should be attributed to the rich enzyme system produced by *R. oligosporus* during fermentation, as several studies have confirmed that the release of phenolic compounds from plant raw materials or food is related to the microbial enzyme system (17, 44–47).

### Antioxidant activity

**FIGURE 7:**
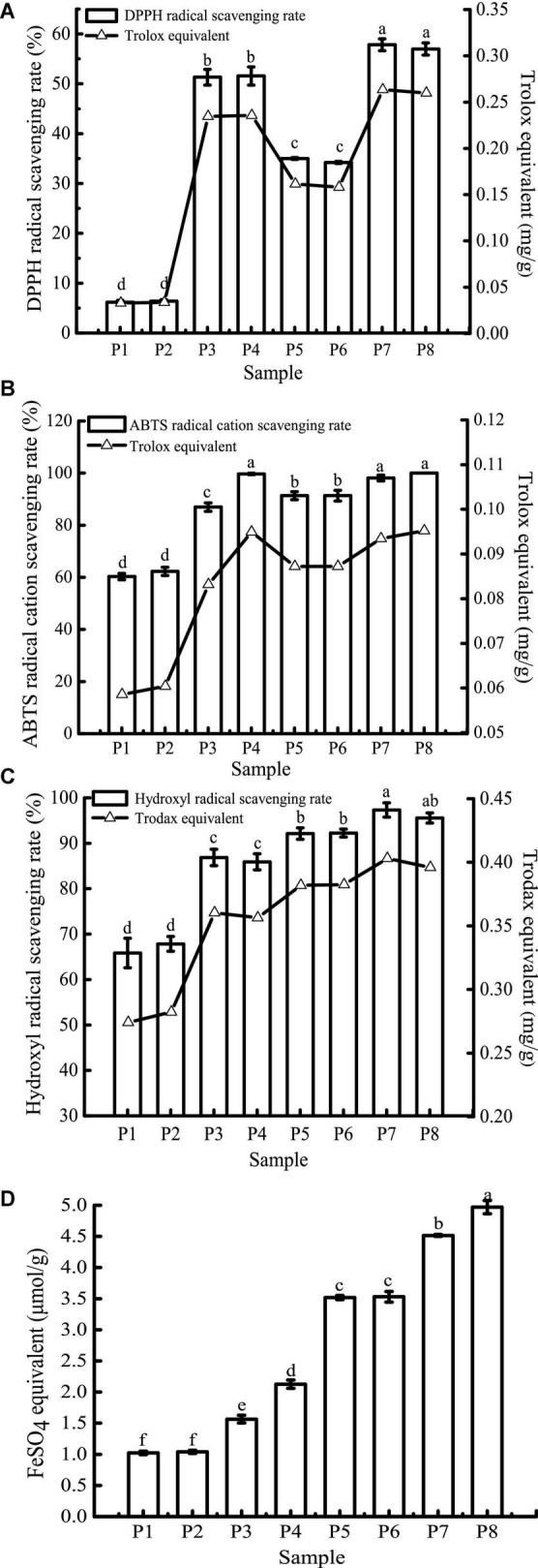
1,1-Diphenyl-2-picrylhydrazyl (DPPH) **(A)**, ABTS^+^ **(B)**, hydroxyl radical scavenging activity **(C)**, and FRAP **(D)** of samples in different simulated digestion stages. Data with different lowercase letters indicate significant differences (*P* < 0.05).

Due to the differences in the mechanisms of antioxidant detection methods, it is difficult to objectively measure the actual antioxidant capacity of a sample using just a single evaluation method (48). In this study, four antioxidant evaluation models, including DPPH, ABTS^+^, hydroxyl radical scavenging ability, and FRAP were used to evaluate the effect of *in vitro* digestion on the antioxidant capacity of the study samples. The results are shown in Figure 7.

The scavenging rates of DPPH, ABTS^+^, and hydroxyl radicals of unfermented black bean samples and black bean tempeh samples were not affected by buccal digestion, but they were significantly increased after gastric digestion (*P* < 0.05), while small intestinal digestion did not further improve the scavenging rates. The change in FRAP was consistent with the free-radical scavenging rate before small intestinal digestion, but it continued to increase significantly after intestinal digestion (*P* < 0.05). There have been many reports on the effect of *in vitro* digestion on the antioxidant capacity of food. For example, compared with that of cooked rice, the oxygen free radical absorption capacity of brown rice and milled rice after *in vitro* digestion was increased by 185.7 and 293.4%, respectively (49). Additionally, *in vitro* digestion improved the antioxidant activity of common beans and pinto beans (50). There are also contradictory results: for instance, Rodriguez-Roque et al. (51) found that *in vitro* gastric digestion significantly increased the total antioxidant activity of soy milk (76%), while small intestinal digestion caused a significant decrease, and they speculated that this decrease may be due to the fact that some substances with antioxidant activity, such as phenolic compounds, are sensitive to alkaline pH. Therefore, these compounds are transformed into compounds with a structural form that exhibits different chemical properties. The plant matrix has a matrix effect on the retention rate of specific phytochemicals (52), hence the food matrix and the composition of phytochemicals may also be responsible for the differences in antioxidant capacity of different food after *in vitro* digestion. In the present study, after *in vitro* digestion, the DPPH, ABTS^+^, hydroxyl radical scavenging rates, and FRAP of unfermented black bean samples reached 8.34, 1.65, 1.30, and 2.09 times the initial values, respectively. In contrast, the corresponding values in black bean tempeh samples only increased 0.63, 0.09, 0.04, and 0.41 times compared to the initial values, respectively. However, in terms of absolute values, at the same digestion stage, the DPPH, hydroxyl radical scavenging ability, and FRAP of unfermented black bean samples were significantly lower than those of black bean tempeh samples (*P* < 0.05), and only the free radical scavenging ability of ABTS^+^ in the unfermented samples was equal to that of tempeh samples after small intestinal digestion, which proved that processing black beans into tempeh could improve their antioxidant activity after consumption.

For DPPH, ABTS^+^, hydroxyl radical scavenging capacity, and FRAP, consistent with the results of TPC, TFC, and PC analysis, *in vitro* digestion caused a significant increase in the antioxidant capacity of both samples. Although the specific assay mechanisms were different, all antioxidant activity resulted in a similar trend. To further clarify the correlation between the content of phenolic compounds and different antioxidant activities, Pearson’s correlation analysis was performed using SPSS 17.0 software. The results are shown in Table 1. DPPH, ABTS^+^, and hydroxyl radical scavenging rates were positively correlated with TPC, TFC, and PC, either significantly or highly significantly. FRAP was positively correlated with TFC and PC (*P* < 0.05). The significant positive correlation indicated that the antioxidant activity was closely related to the release of phenolic compounds during *in vitro* digestion. This was consistent with the conclusion of Zheng et al. (53) that the oxygen radical absorption capacity (ORAC) and the rapid peroxyl radical scavenging capacity of Chinese hawthorn fruit were significantly positively correlated with the amount of total phenolics or flavonoids released. However, Sancho et al. (12) found that although *in vitro* digestion reduced the TPC in the seed coats of black beans and small red beans, as well as the TFC of black bean seed coats, the antioxidant activity of both groups of samples remained unchanged, and the ORAC in the black bean group even increased significantly. This suggests that other soluble compounds present in the digestive fluid, such as simple carbohydrates or amino acids, may interfere with the antioxidant test or the determination of total phenols and that some non-phenolic compounds, such as carotenoids and tocopherols, may also contribute to the antioxidant activity (38, 43).

**TABLE 1:** Pearson’s correlation coefficients among total phenolic content (TPC), total flavone content (TFC), proanthocyanidin content (PC), and antioxidant activity.

	DPPH	ABTS^+^	Hydroxyl radical	FRAP
TPC	0.968**	0.864**	0.779*	0.651
TFC	0.938**	0.830*	0.782*	0.711*
PC	0.912**	0.838*	0.807*	0.793*

*Correlation was significant at the 0.05 level (one-tailed).

**Correlation was significant at the 0.01 level (two-tailed).

## Conclusion

The results of this study confirmed that the proteins of unfermented black beans and black bean tempeh were degraded during *in vitro* digestion, the content of hydrolysates such as soluble proteins and small peptides and the DH of proteins were significantly increased, and phenolic compounds were released, the bioactivity of which was also greatly improved. However, compared with unfermented black beans, the black bean tempeh demonstrated higher bioavailability of proteins and phenolic compounds, and its antioxidant activity was stronger as well. Additionally, its ACE-inhibitory activity was greatly improved following *in vitro* digestion, closing the gap with unfermented black beans. These differences between unfermented black beans and black bean tempeh may be attributed to the biochemical reactions caused by the enzymes produced by *R. oligosporus* during fermentation. Although these *in vitro* results cannot be fully and directly extended to *in vivo* conditions, these data provide preliminary evidence that the nutritional values and potential health benefits of black bean tempeh remain higher than those of unfermented black beans even after consumption.

## Data availability statement

The original contributions presented in this study are included in the article/supplementary material, further inquiries can be directed to the corresponding authors.

## Author contributions

KW and YG designed the research, performed the experiments, and wrote the original draft. JZ, YW, JS, and GN conducted the statistical analysis and the analysis of the results. FZ and XZ designed the experimental scheme, supervised the experiments, and revised the manuscript. All authors contributed to the article and agreed to the published version of the manuscript.
